# Systematic review of the relation between smokeless tobacco and cancer of the pancreas in Europe and North America

**DOI:** 10.1186/1471-2407-8-356

**Published:** 2008-12-01

**Authors:** Zheng Sponsiello-Wang, Rolf Weitkunat, Peter N Lee

**Affiliations:** 1 Philip Morris Products S.A, PMI Research & Development, Neuchâtel, Switzerland; 2 P.N. Lee Statistics and Computing Ltd, Surrey, UK

## Abstract

**Background:**

Recent reviews claiming smokeless tobacco increases pancreatic cancer risk appear not to have considered all available epidemiological evidence; nor were meta-analyses included. We present a systematic review of studies from North America and Europe, since data are lacking from other continents. Risk is also difficult to quantify elsewhere due to the various products, compositions and usage practices involved.

**Methods:**

Epidemiological studies were identified that related pancreatic cancer to use of snuff, chewing tobacco or unspecified smokeless tobacco. Study details and effect estimates (relative risks or odds ratios) were extracted, and combined by meta-analyses.

**Results:**

Nine North American and two Scandinavian studies were identified. Reporting was limited in four studies, so only seven were included in meta-analyses, some providing results for never smokers, some for the overall population of smokers and non-smokers, and some for both.

Giving preference to study-specific estimates for the overall population, if available, and for never smokers otherwise, the random-effects estimate for ever smokeless tobacco use was 1.03 (95% confidence interval 0.71–1.49) based on heterogeneous estimates from seven studies. The estimate varied little by continent, study type, or type of smokeless tobacco.

Giving preference to estimates for never smokers, if available, and overall population estimates otherwise, the estimate was 1.14 (0.67–1.93), again based on heterogeneous estimates. Estimates varied (p = 0.014) between cohort studies (1.75, 1.20–2.54) and case-control studies (0.84, 0.36–1.97). The value for cohort studies derived mainly from one study, which reported an increase for never smokers (2.0, 1.2–3.3), but not overall (0.9, 0.7–1.2). This study also contributed to increases seen for snuff use and for European studies, significant only in fixed-effect analyses.

The studies have various weaknesses, including few exposed cases, reliance in cohort studies on exposure recorded at baseline, poor control groups in some case-control studies, and lack of a dose-response. Publication bias, with some negative studies not being presented, is also possible.

**Conclusion:**

At most, the data suggest a possible effect of smokeless tobacco on pancreatic cancer risk. More evidence is needed. If any risk exists, it is highly likely to be less than that from smoking.

## Background

Smokeless tobacco (ST) is mainly used orally, chewing tobacco and snuff being the major products used in North America and Europe. There are several types of chewing tobacco and snuff, differing in their formulation and in how the tobacco is treated and used [1-3]. In the United States, where finely-cut moist snuff or chewing tobacco is held by users in the gingival buccal area, ST has been an important part of total tobacco consumption for many years. For example, in 2000, ST formed 12.9% of tobacco consumption by weight [4,5], and was used by 4.5% of men and 0.3% of women [6]. Previously, sales of chewing tobacco considerably exceeded those of snuff, but since the 1980s sales of snuff have risen sharply, now exceeding those of chewing tobacco [4,5]. In Sweden, the only other economically developed country where ST forms a major part of tobacco sales (e.g. 53% in 2000), snuff ('snus') is generally placed between the gum and upper lip [2]. The Swedish population has a long history of use of snus, sales of chewing tobacco there being negligible. ST also forms a few per cent of the market in Canada and in other Scandinavian countries [4,5]. ST is also widely used in parts of Central and South-East Asia. Use occurs in various ways, with the tobacco used alone or in combination with other products, such as betel nut quid, slaked lime, areca nut and even snail shells [1,3,7].

In 2008, a report from the European Community Scientific Committee on Emerging and Newly Identified Health Risks (SCENIHR) on the health effects of ST [8] noted that "All STP [ST products] contain nicotine, a potent addictive substance. They also contain carcinogenic tobacco-specific nitrosamines, albeit at differing levels. STP are carcinogenic to humans and the pancreas has been identified as a main target organ." The conclusion relating to the pancreas derived from a review of evidence from eight studies [9-16], and was identical to that in a draft report published in 2007 [17]. No meta-analyses were presented, but it was noted that the pancreas was identified as a main target organ in two Scandinavian studies. When detailing the results for these studies, the data cited were not complete. Notably, for the Norway Cohorts Study by Boffetta *et *al [10], reference was made to a relative risk [RR] for ever use of snuff of 1.67 (95% confidence interval [CI] 1.12–2.50), which was based on results for smokers and non-smokers combined with adjustment for smoking, with no mention made of the RR of 0.85 (95% CI 0.24–3.07) specifically for never smokers. In contrast, for the study of Swedish construction workers by Luo *et al *[11], reference was made to a RR of 2.00 (1.20–3.30) specifically for never smokers, with no reference made to a RR of 0.90 (0.70–1.20), based on results for smokers and non-smokers combined, with adjustment for smoking.

The previous year, in 2007, the International Agency of Research on Cancer (IARC) published a monograph entitled "smokeless tobacco and some tobacco-specific nitrosamines" [2], an earlier indication of its findings having been made available three years before this [18]. The monograph concluded that "there is **sufficient evidence **in humans for the carcinogenicity of smokeless tobacco. Smokeless tobacco causes cancers of the oral cavity and pancreas." The monograph considered the same studies as had SCENIHR [8], but did not consider the Swedish Construction Workers Study [11] and one other study [15] published in 2007, after the IARC working group had completed their deliberations. As in the SCENIHR report, the IARC report did not mention the lack of association of snuff with risk in never smokers in the Norway Cohorts Study [10].

Given the lack of meta-analyses in either report, the apparent failure to consider all the relevant data, and the existence of other studies providing a certain amount of additional information [19-21], we decided to carry out a detailed review of all the available evidence relating ST use to pancreatic cancer in North America and Europe. As in some other reviews of possible effects of ST use (e.g. [22-26], results from other areas (such as South East India), where ST is often mixed with other substances, are not considered. However, it should be noted that no studies of ST and pancreatic cancer conducted outside North America and Europe were referred to in either the IARC monograph 89 [2] or the SCENIHR report [8].

## Methods

### Study identification and selection

Relevant studies were identified by literature searches through December 2007 using EMBASE, MEDLINE and references listed in the identified publications. The search was not limited by period or language. The main searches were based on combinations of the terms "smokeless tobacco", "chewing tobacco", "snuff", and "snus" for exposure and "pancreatic cancer" for outcome. Study selection was restricted to epidemiological reports which presented data on pancreatic cancer mortality or morbidity associated with use of snuff, chewing tobacco or unspecified ST.

All reports had to satisfy the following **Inclusion criteria**: based on research on humans, of cohort or case-control design, any form of pancreatic cancer as the outcome, and chewing tobacco, orally used moist snuff or unspecified smokeless tobacco as the exposure. The reports also had to satisfy the **Exclusion criteria**: sample included in a more complete or recent study, conducted in an Asian population, no control group or inappropriate design (case report, qualitative study or review/meta-analysis).

### Data extraction

From each study, details were extracted of the study design, location and timing, and of the potential confounding variables considered. Where available, estimates of the RR or odds ratio [OR] associated with ST use, including dose-response data, were extracted together with their associated CI. On occasion, the effect estimates (RR or OR) were calculated from data provided in the source publication. Attention was restricted to estimates where the two ST use groups being compared were both never smokers, both current smokers, both former smokers, or both the overall population of smokers and non-smokers combined.

### Meta-analyses

Fixed-effect and random-effects meta-analyses were conducted as described by Fleiss and Gross [27] with the output showing the combined effect estimate (with 95% CI) from each analysis, together with the results of the chisquared test of homogeneity and the I^2 ^statistic [28]. For selected meta-analyses, a forest plot is also shown. Each estimate is shown as a rectangle, the area of which is proportional to its weight. The CI is indicated by a horizontal line. The data are plotted on a logarithmic scale so that the estimate is centred within the CI. Also shown in the plot are the actual values of the estimate and its CI and weight. Results from a random effects meta-analysis are also shown. The combined estimate is presented as a diamond with the width corresponding to the CI, and the estimate as the centre of the diamond.

As will become apparent later, data in a form suitable for meta-analyses were only available for seven studies, with some only reporting results for never smokers and some only reporting results for the overall population of smokers and non-smokers combined, and some both. Accordingly, two major sets of estimates were selected for meta-analysis. One was based on overall population estimates where available, otherwise on estimates for never smokers. The other was based on the never smoker estimates where available, otherwise on estimates for the overall population. For each set, preference was given to estimates comparing ever ST use with never ST use, as these were always available, while alternatives (e.g. comparing current and never ST use) rarely were. Where separate estimates were available for chewing tobacco and for snuff, results for chewing tobacco were used in the main meta-analyses, though alternative analyses preferring estimates for snuff were also carried out. Meta-analyses were also conducted for subdivisions of the studies by continent, type of ST, study type (prospective or case-control) and smoking group.

## Results

Table 1 summarizes details of the four cohort studies [9-11,19] for which results have been reported relating ST use to pancreatic cancer incidence or mortality. The sources cited give the most recent (and comprehensive) publications, although for two of these studies earlier publications [29-32] provide more limited results from a shorter follow-up. The studies are all in males, except for a very small proportion of females in the US veterans study [33]. Two of the studies were conducted in the USA and concern unspecified ST. The other two were conducted in Scandinavia and concern snuff use. The largest study, of US veterans, has only provided limited results, for fifteen years of follow-up, in an abstract [19]. The other three studies involve follow-up periods of 20 to 36 years. All of the cohort studies use exposure data as recorded at baseline.

**Table 1:** Cohort studies of smokeless tobacco and pancreatic cancer

Study details	US Veterans Study	Lutheran Brotherhood Study	Norway Cohorts Study	Swedish Construction Workers Study
				

Source^a^	Winn et al [19]^b^	Zheng et al [9]^c^	Boffetta et al [10]^d^	Luo et al [11]

Country	USA	USA	Norway	Sweden

Population	US veterans insurance policyholders	Lutheran brotherhood insurance policyholders^e^	General population samples and relatives of Norwegian migrants to the USA	Construction workers

Baseline survey	1954	1966	1964, 1967	1978–1992

Sex	> 99.5% Male	Male	Male	Male

Follow-up	1954–1969	1966–1986	1966–2001	1978–2004

Sample size^f^	Approx 300,000	17,633	10,136	279,897

Endpoint	Mortality	Mortality	Incidence^g^	Incidence

Data on ST use	ST unspecified	ST unspecified	Snuff	Snuff

Adjustment factors^h^	Age	Age, smoking, alcohol	Age, smoking	Age, smoking, body mass index

^a ^The source given is for the latest follow-up

^b ^The source is an abstract. Some of the study information about the US Veterans Study was obtained from other sources [33]

^c ^Page 103 of IARC Monograph 37 [30] summarizes 15 year follow-up results from this study based on two abstracts [29,32]

^d ^11 year follow-up results from this study are reported on page 103 of IARC Monograph 37 [30] based on two abstracts [29,32], while 14 year follow-up results are given by Heuch *et al *[31]

^e ^White

^f ^Numbers used in specific analyses may be less than this

^g ^Cases diagnosed on the basis of a clinical examination or death certificate only were excluded

^h ^Adjustment factors used in analysis

Table 2 summarizes details of seven case-control studies [12-16,20,21], all conducted in the USA or Canada. Results for ST are available for males and females combined in four studies [14,15,20,21] and for males only in the other three studies [12,13,16]. The studies vary as to whether they report results for chewing tobacco and snuff separately [13,15,20], for chewing tobacco only [12,21], or for combined ST use based on chewing tobacco or snuff [14,16]. Of the seven studies, all concern incident cancer, except for one [21] that makes no such distinction. In humans, the great majority of pancreatic cancers are exocrine adenocarcinomas, endocrine (islet-cell) carcinomas being far less common, so that the stated restrictions to "carcinoma of the exocrine pancreas" in one study [14] or to "pancreatic adenocarcinoma" in another [15] should be of little consequence. One study also included cancers of the bile duct or gall bladder [21], but these form only a small proportion of the total. Three studies used population based controls [12,14,21], while two used hospital patients [13,20] and one hospital visitors [15]. One study [16] was based on the Third National Cancer Survey and compared pancreatic cancer cases with cancers considered not to be associated with smoking. One study [12] used only surrogate respondents, while two studies [20,21] used some. The numbers of cases studied varied from 113 [16] to 808 [15].

**Table 2:** Case-control studies of smokeless tobacco and pancreatic cancer

Study Details	Third National Cancer Survey Study	Louisiana Study	Washington State Study	Quebec Study
				

Source	Williams et al [16]	Falk et al [20]	Farrow et al[12]	Ghadirian et al [21]

Country	USA^a^	USA	USA	Canada

Timing	1969–1971	1979–1983	1982–1986	1984–1988

Sex	Males^b^	Males and females	Males^c^	Males and females

Cases	Incident, pancreatic cancer	Incident, pancreatic cancer	Incident, pancreatic cancer	Diagnosed, pancreatic, bile duct or gall bladder cancer

Controls	Incident, cancers not associated with smoking	Hospital, without diet-altering chronic disease	Population, random-digit dialling	Population, random-digit dialling

Matching	None	Age, sex, race	Age, area	Age, sex, area

Surrogate respondents	None	> 50% (cases), 13% (controls)	100% (cases), 100% (controls)	75% (cases), 17% (controls)

Cases^d^	113^e^	363	168	179

Controls^d^	2074^e^	1234	195	239

Data on ST use^f^	Chewing or snuff	Chewing, snuff	Chewing	Chewing

Adjustment factors^g^	Age, race, smoking	Unstated	Race, education	Age, sex, smoking, education, proxy response

				

	Nine Hospital Study	Fifteen County Study	Texas Study	
	
				
	
Source	Muscat et al [13]	Alguacil and Silverman [14]	Hassan et al [15]	
	
Country	USA^h^	USA^i^	USA	
	
Timing	1985–1993	1986–1989	2000–2006	
	
Sex	Males^b^	Males and females	Males and females	
	
Cases	Incident, pancreatic cancer	Incident, exocrine pancreatic cancer	Incident, pancreatic adenocarcinoma	
	
Controls	Hospital, diseases not associated with smoking	Population, random-digit dialling^j^	Hospital visitors, healthy, no cancer history	
	
Matching	Age, sex, race, hospital, year of diagnosis	Age, sex, race	Age, sex, race	
	
Surrogate respondents	None	None	None	
	
Cases^d^	290^e^	154	808	
	
Controls^d^	572^e^	844	808	
	
Data on ST use^f^	Chewing, snuff	Chewing or snuff	Chewing, snuff	
	
Adjustment factors^g^	None	Age, sex, race, site, cigar smoking	Age, sex, race, smoking, diabetes, alcohol, education, area, marital status	

^a ^Nationwide

^b ^Females were also studied, but results were only available for males

^c ^Only married men were included

^d ^Numbers used in specific analyses may be less than this

^e ^Numbers are of males

^f ^"Chewing or snuff" implies that results were only available for combined ST use; "chewing, snuff" implies they were separately available

^g ^Adjustment factors used in analysis

^h ^Conducted in four hospitals in New York, two in Philadelphia, and one each in Chicago, and Detroit

^i ^Conducted in two counties in Atlanta, three in Detroit and ten in the state of New Jersey

^j ^Controls aged 65–79 were drawn from Medicare and Medicaid service rosters

### Effect estimates

Table 3 summarizes the results from the 11 studies. For four of the studies [12,19-21], RRs with CIs were not available, so the studies could not be included in the meta-analyses. Of those four studies, the Louisiana [20], Washington State [12] and Quebec [21] studies reported no indication of an increased risk of pancreatic cancer associated with ST use, but the US Veterans Study [19] did indicate an elevated RR of 1.65, although the abstract made no statement about significance. Of the other seven studies, the Norway cohorts [10] and Swedish construction workers [11] studies reported a significant excess in some analyses but not others, while the Nine Hospital Study [13] reported a near significant increase in one analysis. The Third National Cancer Survey [16] reported a decrease that we estimate as significant. The other three studies [9,14,15] reported no significant increase or decrease.

**Table 3:** RR/OR of pancreatic cancer associated with smokeless tobacco use

Study^a^	ST use		Smoking	Sex	RR/OR			
				
	Type^b^	Exposure^c^			No.	Cases^d^	Estimate (95% CI)	Notes
								

**Cohort studies**					**RR**			

US Veterans [19]	ST	Use	Never	M	1	NA	1.65	e, f

Lutheran Brotherhood [9]	ST	Ever	Any	M	2	16	1.70(0.90–3.10)	

Norway Cohorts [10]	Snuff	Ever	Any	M	3	45	1.67(1.12–2.50)	g

		Former	Any		4	18	1.80(1.04–3.09)	

		Current	Any		5	27	1.60(1.00–2.55)	

		Ever	Never		6	3	0.85(0.24–3.07)	

		Ever	Former		7	14	1.37(0.59–3.17)	

		Ever	Current		8	28	1.86(1.13–3.05)	

Swedish Construction Workers [11]	Snuff	Ever	Any	M	9	NA	0.90(0.70–1.20)	

		Ever	Never		10	20	2.00(1.20–3.30)	

		Former	Never		11	2	1.40(0.40–5.90)	

		Current	Never		12	18	2.10(1.20–3.60)	

**Case-control studies**					**OR**			

Third National Cancer Survey [16]	ST	Ever	Any	M	13	3	0.29(0.09–0.92)	h

Louisiana [20]	Chewing	Use	Any	M+F	14	NA	"No excess risk"	

	Snuff	Use	Any		15	NA	"No excess risk"	

Washington State [12]	Chewing	Ever	Any	M	16	NA	0.80 (Not sig.)	i

Quebec [21]	Chewing	Use	Any	M+F	17	NA	"Not associated with increased risk"	

Nine Hospital [13]	Chewing	Ever	Never^j^	M	18	6	2.82(0.95–9.39)	k

	Snuff	Ever	Any		19	2	1.32(0.22–7.93)	h

Fifteen County [14]	ST	Ever	Never	M+F	20	5	1.10(0.40–3.10)	l

Texas [15]	Chewing	Ever	Any	M+F	21	34	0.70(0.40–1.10)	

		Ever	Ever		22	24	0.70(0.40–1.20)	

		Ever	Never		23	10	0.60(0.30–1.40)	

	Snuff	Ever	Any		24	18	0.60(0.30–1.10)	

		Ever	Ever		25	14	0.70(0.30–1.40)	

		Ever	Never		26	4	0.50(0.10–1.50)	

^a ^Fuller details of the studies are given in Tables 1 and 2

^b ^ST implies smokeless tobacco unspecified or combined snuff use or chewing

^c ^Ever, former and current ST are compared with never ST. Use indicates timing not given and comparison is with non use

^d ^Cases in ST users as defined, NA = not available

^e ^The abstract suggests the results are for never smokers, but this is not totally clear. The population included < 0.5% females. In earlier reports from this study [30] RRs were reported of 3.3 (statistically significant) for former ST users, and of 2.1 (not significant) for current ST users, based on, respectively, 7 and 5 cases in the exposed group

^f ^Confidence intervals could not be calculated

^g ^In earlier reports the risk of histologically-reported pancreatic cancer in regular ST users was stated to be 2.2 ("significant") [30] and 2.9 (trend p = 0.06) [31]

^h ^Estimated from data provided

^i ^Additional adjustment for age and dietary factors did not materially affect the odds ratios

^j ^Includes long term (> 10) years quitters

^k ^Personal communication from the author

^l ^The authors also reported an adjusted OR of 1.4 (0.5–3.6) for the comparison (among never cigarette smokers) of ever used ST, but may have used pipe or cigar versus never used ST, pipe or cigar

**Figure 1:**
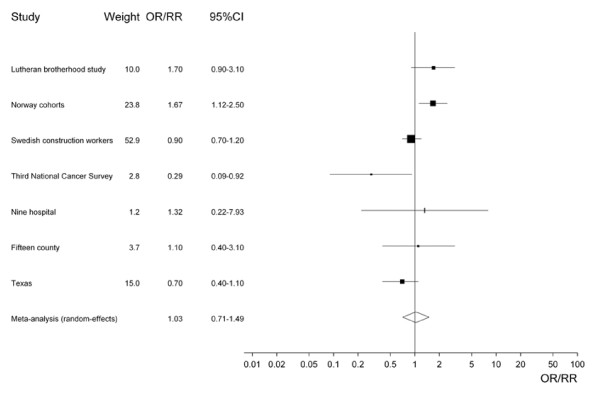
Forest plot of study-specific effect estimates and 95% CIs, using overall population estimates where available.

Table 4 presents the results of the meta-analyses using overall population estimates where available, and estimates for never smokers if not. All the overall population estimates were adjusted for smoking. The principal analysis (see also Figure 1), based on RR/OR estimates numbered 2, 3, 9, 13, 19, 20 and 21 in Table 3, shows no evidence of an effect of ST use, since both the fixed-effect estimate (1.04, 95% CI 0.86–1.25) and the random-effects estimate (1.03, 0.71–1.49), were only slightly in excess of 1.00 and not statistically significant. However, there is evidence of heterogeneity (chisquared 15.95 on 6 d.f., p = 0.014). The lack of a statistically significant association is unaffected by using estimates for snuff rather than chewing tobacco in the two studies [13,15] where there was a choice.

**Table 4:** Meta-analyses of overall population estimates of pancreatic cancer risk associated with ST, using estimates for never smokers if overall population estimates are not available^a^

Meta-analysis	Estimates Included	Fixed-effect RR (95% CI)	Random-effects RR (95% CI)	Heterogeneity		
				
				Chisquared (d.f.)	I^2^	p
						

All studies	2,3,9,13,19,20,21	1.04(0.86–1.25)	1.03(0.71–1.49)	15.95 (6)	62.4	0.014

All, preferring snuff to chewing^b^	2,3,9,13,19,20,24	1.04(0.86–1.27)	1.01(0.68–1.50)	16.35 (6)	63.3	0.012

USA or Canada	2,13,19,20,21	0.92(0.65–1.29)	0.89(0.50–1.60)	8.97 (4)	55.4	0.062

Sweden or Norway^c^	3,9	1.09(0.87–1.36)	1.20(0.66–2.20)	6.28 (1)	84.1	0.012

Cohort	2,3,9	1.15(0.93–1.42)	1.31(0.82–2.11)	8.03 (2)	75.1	0.018

Case-control^d^	13,19,20,21	0.70(0.46–1.05)	0.70(0.43–1.13)	3.44 (3)	12.7	0.329

Chewing tobacco	18,21	0.88(0.55–1.40)	1.27(0.33–4.92)	4.76 (1)	79.0	0.029

Snuff	3,9,19,24	1.03(0.83–1.27)	1.02(0.64–1.64)	9.26 (2)	67.6	0.026

ST unspecified	2,13,20	1.14 (0.70–1.84)	0.89(0.33–2.40)	6.94 (2)	71.2	0.031

Overall estimates exclusively	2,3,9,13,19,21	1.03(0.85–1.25)	1.01(0.67–1.54)	15.93 (5)	68.6	0.007

Excluding Third National Cancer Survey Study[16]	2,3,9,19,20,21	1.07(0.89–1.29)	1.13(0.80–1.59)	11.22 (5)	55.4	0.047

^a ^See Table 3 for the individual study estimates

^b ^Where estimates for both snuff and chewing are available

^c ^There was no significant variation by continent (chisquared = 0.70 on 1 d.f., p = 0.404)

^d ^There was significant variation by study type (chisquared = 4.48 on 1 d.f., p = 0.034)

The significant heterogeneity of the seven study-specific estimates cannot readily be explained. Estimates do not vary significantly by continent and are similar for chewing tobacco, snuff and unspecified ST. Excluding results for never smokers had little effect on the estimates. Although estimates were significantly (p = 0.034) higher for cohort studies than for case-control studies, heterogeneity was still evident in the cohort studies, and indeed in all of the analyses shown in Table 4 except for the case-control studies. No single study contributes notably to the heterogeneity. Excluding the Third National Cancer Survey Study [16], which used a design that may be inappropriate (see discussion), and unusually reported a significant reduction in risk associated with ST use, still leaves the overall estimate not significantly different from 1.00 and reduces the heterogeneity only slightly.

**Figure 2:**
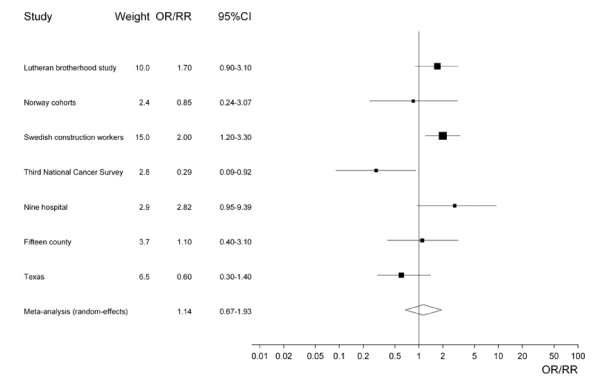
Forest plot of study-specific effect-estimates and 95% CIs, using estimates for never (or non-current) smokers where available.

Table 5 presents the results of the meta-analyses using estimates for never smokers where available, and overall population estimates if not. Generally, these analyses are more suggestive of a possible effect than the results shown in Table 4. For the principal analysis (see also Figure 2), based on estimates 2, 6, 10, 13, 18, 20 and 23, the fixed-effect estimate is near significant (1.32, 95% CI 0.98–1.77). However there is substantial heterogeneity between studies (chisquared 16.06 on 6 d.f., p = 0.013), and the random-effects estimate is lower and not significant (1.14, 0.67–1.93). There is no real indication of an effect of ST in case-control studies, in studies in North America, or in studies of chewing tobacco or unspecified ST use, though again estimates were heterogeneous. There is, however, more indication of an effect in cohort studies, in studies in Sweden or Norway, and in studies of snuff, where fixed-effect estimates of the RR associated with ST exposure are all significant or borderline significant (cohort 1.75 95% CI 1.20–2.54, Sweden or Norway 1.78, 1.11–2.85, and snuff 1.54, 1.00–2.37). All three estimates are strongly affected by the major contribution of the RR estimate of 2.00 (1.20–3.30) for the Swedish Construction Workers Study [9], which for reasons noted in the discussion may substantially overestimate any true relationship. This study contributes 54.7% of the weight for cohort studies, 86.4% of the weight for studies in Sweden and Norway, and 72.6% of the weight for studies of snuff. Though none of these three analyses themselves shows significant heterogeneity, the marked heterogeneity for the "all studies" meta-analysis in Table 5 suggests that random-effects estimates may possibly be more appropriate. If so, only the estimate for cohort studies remains significant. This combined estimate of 1.75 (1.20–2.54) is based on three individual study estimates, that noted above from the Swedish Construction Workers Study [11] of 2.00 (95% CI 1.20–3.30, weight 15.02), an estimate of 0.85 (0.24–3.07, 2.37) from the Norway Cohort Study [10] and an estimate of 1.70 (0.90–3.10, 10.05) from the Lutheran Brotherhood Study [9], this final estimate not being specifically for never smokers. A significant fixed-effect estimate (1.46, 1.08–1.99) is also seen when the Third National Cancer Survey Study [16] is excluded from consideration on the grounds of its study design. However the random-effects estimate is not significant, and again the Swedish Construction Workers Study [11] contributes substantially (37.1% of the weight) to the analysis.

**Table 5:** Meta-analyses of estimates of pancreatic cancer risk associated with ST for never smokers, using overall population estimates if never smoker estimates are not available^a^

Meta-analysis	Estimates Included	Fixed-effect RR (85% CI)	Random-effects RR (95% CI)	Heterogeneity		
				
				Chisquared (d.f.)	I^2^	P
						

All studies	2,6,10,13,18,20,23	1.32(0.98–1.77)	1.14(0.67–1.93)	16.06 (6)	62.6	0.013

All preferring snuff to chewing^b^	2,6,10,13,19,20,26	1.35(0.98–1.86)	1.08(0.64–1.82)	12.31 (6)	51.2	0.055

USA or Canada	2,13,18,20,23	1.08(0.73–1.58)	1.00(0.50–2.02)	11.92 (4)	66.4	0.018

Sweden or Norway^c^	6,10	1.78(1.11–2.85)	1.61(0.77–3.34)	1.50 (1)	33.2	0.221

Cohort	2,6,10	1.75 (1.20–2.54)	1.75(1.20–2.54)	1.51 (2)	0.0	0.470

Case-control^d^	13,18,20,23	0.81(0.49–1.32)	0.84(0.36–1.97)	8.48 (3)	64.6	0.037

Chewing tobacco	18,23	0.97(0.51–1.84)	1.22(0.27–5.55)	4.83 (1)	79.3	0.028

Snuff	6,10,19,26	1.54(1.00–2.37)	1.25(0.64–2.44)	4.54 (3)	33.9	0.209

ST unspecified	2,13,20	1.14(0.70–1.84)	0.89(0.33–2.40)	6.94 (2)	71.2	0.031

Never smoking exclusively	6,10,18,20,23	1.39(0.98–1.99)	1.28(0.71–2.30)	8.80 (4)	54.5	0.066

Excluding Third National Cancer Survey Study [16]	2,6,10,18,20,23	1.46(1.08–1.99)	1.38(0.88–2.15)	9.10 (5)	45.0	0.105

^a ^See Table 3 for the individual study estimates

^b ^Where estimates for both snuff and chewing are available

^c ^There was no significant variation by continent (chisquared = 2.64 on 1 d.f., p = 0.104)

^d ^There was significant variation by study type (chisquared = 6.07 on 1 d.f., p = 0.014)

The two Scandinavian studies [10,11] reported results for current and former ST use (see Table 3). In both studies the risk estimate for quitters is similar to that for continuing users.

Table 6 shows the limited data available on a possible dose relationship between ST use and risk of pancreatic cancer. The three studies [11,15,16] each provide quite similar risk estimates for high and low usage, albeit based on small numbers of exposed cases at each usage level. Not shown in Table 6 are the dose-response results from the Fifteen County Study [14], which did report a significant trend (p = 0.04) with ounces/week ST ever used (though not with years used), based on seven ST-exposed cases. This was based on an analysis that may be biased by the fact that the exposed group, but not the reference group, may have smoked pipes and cigars. Therefore, it did not satisfy our criteria for consideration in analysis. In any case the trend, based on ORs of 1.00 (reference), 0.30 (95% CI 0.04–2.50) for ≤ 2.5 ounces ST per week, and 3.50 (95% CI 1.10–10.60) for > 2.5 ounces ST per week, was partly due to a low OR in the low exposure group. It is clear that the overall data have not demonstrated a dose-response relationship.

**Table 6:** Dose-response data for smokeless tobacco and pancreatic cancer

Study^a^	Smoking	Sex	ST use		RR/OR	
				
			Type	Exposure	Cases	Estimate (95% CI)
						

Swedish Construction Workers [11]	Never	M	Snuff	Never	63	1.00 (reference)

				1–9 g/day	6	1.90 (0.80–4.30)

				10+ g/day	13	2.10 (1.10–3.80)

Third National Cancer Survey [16]	Any	M	ST^b^	Never	88	1.00 (reference)

				Low^c^	2	0.31 (0.08–1.27)^d^

				High^c^	1	0.25 (0.04–1.89)^d^

Texas [15]	Any^e^	M+F	Chewing	Never	774	1.00 (reference)

				Low/moderate^f^	22	0.60 (0.30–1.20)

				High	12	0.60 (0.30–1.20)

			Snuff	Never	790	1.00 (reference)

				Low/moderate^f^	9	0.60 (0.20–1.50)

				High^g^	9	0.60 (0.20–1.30)

^a ^Fuller details of the studies are given in Tables 1 and 2

^b ^Chewing or snuff

^c ^The exposed population was divided into approximately equal numbers by the product of consumption x years of use

^d ^CI were estimated from data provided

^e ^The source also provides results for ever cigarette smokers – chewing 1.00, 0.70 (0.40–1.40), 0.50 (0.20–1.50), snuff 1.00, 0.80 (0.30–2.10), 0.50 (0.20–1.50), and also for never cigarette smokers – chewing 1.00, 0.60 (0.20–2.30), 0.60 (0.20–1.70), and snuff 1.00, 0.00, 0.60 (0.20–2.10)

^f ^Low or moderate intake: ≤ 20 time-years (times/day x years of use)

^g ^High intake: > 20 time-years

## Discussion

When preference is given to overall population smoking-adjusted estimates of pancreatic cancer risk associated with ST, on the basis that these provide greater power to detect a possible association, the meta-analyses (Table 4) show no clear evidence of any increase in risk associated with ST use. In contrast, when preference is given to risk estimates for never smokers, on the basis that these avoid possible confounding by smoking, the meta-analyses (Table 5) show more indication of an increase in risk. While no increased risk is demonstrated in studies in North America or in case-control studies (all of which were in North America), there is some evidence of an increased risk in studies in Sweden or Norway and in the cohort studies. This is also suggested by an analysis excluding the unusually low estimate from the Third National Cancer Survey [16], a study which in any case suffers from a weakness we describe later.

Of the three cohort studies contributing to the analysis in Table 5, one conducted in the USA, one in Sweden and one in Norway, the Norway Cohorts Study [10] shows no increase at all (RR 0.85, 95% CI 0.24–3.07) in never smokers, but the estimate is based on only three pancreatic cancer cases in snuff users, while the Lutheran Brotherhood Study [9] shows a non-significant increase (1.70, 0.90–3.10) based on 16 cases in ST users in an analysis for smokers and non-smokers combined adjusted for smoking (and also age and alcohol).

The major contributor to the observed increase derives from the recently reported Swedish Construction Workers Study [11], where the RR was 2.00 (1.20–3.30), based on 20 cases of pancreatic cancer in snuff-using never smokers. In considering this report by Luo *et al*, a number of points should be made.

First, the study was based on follow-up until the end of 2004, based on exposure information obtained at the first health check-up of those cohort members with at least one visit in the 1978–1992 period. The authors reported that a proportion of subjects classified on their first visit as never smoking were recorded in a later visit as having smoked, and that this proportion was greater in subjects who initially reported use of snus (12% vs 7%). They stated (based on an analysis not reported in detail) that bias due to this misclassification would have had little effect on the risk estimates. However, the estimated bias may have been greater, had account been taken of the possibility of subjects taking up smoking later in the follow-up period, when data were not collected.

Second, as pointed out by Rodu [34], the authors excluded workers enrolled in their cohort from 1971 to 1975 because of "ambiguities" in questionnaire coding, despite an earlier report on snus and cardiovascular disease from this cohort [35] being based only on workers enrolled in the years excluded by Luo *et al *(1971 to 1974).

Finally, the study provided no indication of an increase in the overall population of smokers and non-smokers combined, with the RR adjusted for age, smoking and body mass index estimated as 0.90 (95% CI 0.70–1.20). Though this estimate would have been based on considerably more snuff-using pancreatic cancer cases, Luo *et al *tended to dismiss this finding, and placed far more reliance on the estimate for never smokers, as this avoids residual confounding by smoking. They noted that "previous evidence, reinforced by observed data in the present study (not shown), suggests that individuals who combine smoking with snus use smoke less and might increase their overall chances of subsequent abstinence, compared with those who only smoke". While some bias may be present for this reason, it seems implausible that it could possibly explain the huge difference between the estimates for ever vs. never snuff use of 0.90 (0.70–1.20) for smokers and non-smokers combined and that of 2.00 (1.20–3.30) for never smokers. The same publication [11] also reports corresponding results for lung cancer of 0.70 (0.60–0.70) for smokers and non-smokers combined and of 0.80 (0.50–1.30) for never smokers. Given that smoking has a much greater effect on lung cancer risk than on pancreatic cancer risk [36,37], any residual confounding in the estimated ever vs. never snuff use for smokers and non-smokers combined should be substantially greater for lung cancer than for pancreatic cancer. The lack of any marked difference between the two estimates for lung cancer suggests residual confounding cannot explain more than a minor part of the difference between the two estimates for pancreas cancer. The high estimate in snuff users for never smokers in the Swedish Construction Workers Study seems therefore likely to be due to other reasons, including possibly chance.

No epidemiological study is perfect, and there are weaknesses in those studies that showed no evidence of an association of ST use with pancreatic cancer (see below). However, given the reliance that has been placed on the Swedish Construction Workers Study in a recent review ([8]), it is not inappropriate to have focused on some particular weaknesses of this study, as well as to have pointed out that estimates for other comparisons in this study do not show an association of ST with increased pancreatic cancer risk.

Considering the evidence as a whole, there are a number of limitations which should be pointed out. These include the small number of exposed cases, with the main analysis for never smokers based on a total of only 63 exposed cases from the seven studies providing data, limited control of confounding with, for example, no study at all having taken diet into account (other than alcohol) in their analyses of risk from ST, and the very limited reporting of results relating to ST use for a number of the studies, either because only an abstract was available [19], or because ST was not a major issue in the publication [9,12,13,15,20].

Individual study weaknesses are also an issue. The Louisiana Study and the Quebec Study [20,21] used surrogate respondents markedly more often for cases than for controls. The analysis of the Third National Cancer Survey data [16] included in its control group of cancers not associated with smoking some (e.g. kidney cancer, stomach cancer and leukaemia) for which there is now evidence of a moderate relationship [37]. The Louisiana Study [20] used a control group where exclusions were based on whether the conditions were suspected of altering diet, rather than on grounds of an association with tobacco use. Reliance, in the four cohort studies [9-11,19], all involving follow-up of at least 15 years, on exposure data recorded at baseline, as already noted for the Swedish Construction Workers Study [11], is also a problem. In regard to the Norway Cohorts Study [10], this issue has also been referred to by Ramström [38], who comments on the considerable changes in snus use and smoking over the long follow-up period.

Other general points affecting the overall interpretation are the lack of evidence of any dose-response relationship, and possible publication bias, not only due to failure to publish data that show no effect, but also to failure to present data in a form suitable for meta-analysis where no relationship is seen [12,20,21].

While it remains possible that ST may increase the risk of pancreatic cancer, more evidence is clearly needed to demonstrate this. In any event, any excess risk is highly likely to be less than that associated with active smoking, where there is clear evidence that the risk increases with daily cigarette consumption and the number of years of smoking, and RRs range from three up to five at the highest levels of smoking [36,37].

Our conclusions are not in line with those of the SCENIHR report [8] or of IARC Monograph 89 [2]. The SCENIHR report, as already noted, was limited by emphasis on specific results from the Norway Cohorts Study [10] and from the Swedish Construction Workers Study [11] without citing other relevant results from these two studies. IARC Monograph 89 [2] did not include some recent studies [11,15] and based its conclusions on four studies; one of these [9] did not report a significant relationship with ST use, one of these [13] reported a marginally significant association with chewing tobacco but not snuff, one of these [14] reported no overall association with ST use, but a marginally significant trend with amount used in an analysis that, as explained above, is biased, and one of these [10] reported an association with snuff use in an analysis which effectively assumed that such use would not change over a follow-up period of over 30 years, and also found no association with snuff use in never smokers. Both reviews omitted some relevant references [19-21] and neither included any meta-analyses. Taking into account the limitations of the SCENIHR report [8] and of IARC Monograph 89 [2], and also the weaknesses of the available data, we consider that our analysis more accurately reflects the present evidence regarding the risk of pancreatic cancer associated with smokeless tobacco use, and indicates that an effect has not been demonstrated.

## Conclusion

The available data relating pancreatic cancer to ST use are limited, and relatively weak. Random-effects meta-analyses based on evidence from seven studies do not show a significant relationship of ST use with pancreatic risk, whether (a) attention is restricted specifically to estimates for never smokers (RR 1.28, 95% CI 0.71–2.30, n = 5), (b) estimates for never smokers are used where available and overall population estimates used otherwise (1.14, 0.67–1.93, n = 7), or (c) overall population estimates are used where there is a choice (1.03, 0.71–1.49, n = 7). While some subgroup analyses based on the second set of estimates seem to suggest a possible association, all of these are heavily dependent on the contribution of one specific relative risk estimate from one study with known weaknesses [11].

The data, taken as a whole, are no more than suggestive of a possible effect. More evidence is needed to determine if a true relationship exists. Any risk that may exist is highly likely to be less than that associated with active smoking.

## Abbreviations

CI: 95% confidence interval; IARC: International Agency for Research on Cancer; OR: odds ratio; PMI: Philip Morris International; RR: relative risk; SCENIHR: Scientific Committee on Emerging and Newly Identified Health Risks; ST: smokeless tobacco; STP: smokeless tobacco products.

## Competing interests

ZS-W and RW work for Philip Morris International (PMI), R&D. Both receive their salary from PMI and both own shares in Altria, the holding company of PMI. PNL, founder of P.N. Lee Statistics and Computing Ltd., is an independent consultant in statistics and an advisor in the fields of epidemiology and toxicology to a number of tobacco, pharmaceutical and chemical companies.

## Authors' contributions

PNL completed an unpublished review and meta-analysis of ST and various cancers in March 2007 and later that year prepared a further unpublished review and meta-analysis specifically for pancreatic cancer. During that year ZS-W and RW carried out their own meta-analysis of this evidence. After discussion, it was decided to publish a paper jointly. The text and meta-analyses were drafted by PNL, with the other two authors helping to check the meta-analyses and clarify the text. The figures were prepared by ZS-W. All authors read and approved the final manuscript.

## Pre-publication history

The pre-publication history for this paper can be accessed here:



## References

[B1] International Agency for Research on Cancer (1986). Tobacco smoking [IARC Monographs on the evaluation of the carcinogenic risk of chemicals to humans].

[B2] International Agency for Research on Cancer (2007). Smokeless tobacco and some tobacco-specific N-nitrosamines [IARC Monographs on the evaluation of carcinogenic risks to humans].

[B3] Bolinder G, Bolliger CT, Fagerström KO (1997). Smokeless tobacco – a less harmful alternative?. The tobacco epidemic.

[B4] Forey B, Hamling J, Lee P, Wald N, (Eds) (2002). International Smoking Statistics A collection of historical data from 30 economically developed countries.

[B5] Forey B, Hamling J, Hamling J, Lee P, (Eds) (2006). International Smoking Statistics A collection of historical data from 30 economically developed countries Web edition.

[B6] Nelson DE, Mowery P, Tomar S, Marcus S, Giovino G, Zhao L (2006). Trends in smokeless tobacco use among adults and adolescents in the United States. Am J Public Health.

[B7] Critchley JA, Unal B (2003). Health effects associated with smokeless tobacco: a systematic review. Thorax.

[B8] Scientific Committee on Emerging and Newly Identified Health Risks (SCENIHR) (2008). Health effects of smokeless tobacco products.

[B9] Zheng W, McLaughlin JK, Gridley G, Bjelke E, Schuman LM, Silverman DT, Wacholder S, Co-Chien HT, Blot WJ, Fraumeni JF (1993). A cohort study of smoking, alcohol consumption, and dietary factors for pancreatic cancer (United States). Cancer Causes Control.

[B10] Boffetta P, Aagnes B, Weiderpass E, Andersen A (2005). Smokeless tobacco use and risk of cancer of the pancreas and other organs. Int J Cancer.

[B11] Luo J, Ye W, Zendehdel K, Adami J, Adami H-O, Boffetta P, Nyrén O (2007). Oral use of Swedish moist snuff (snus) and risk of cancer of the mouth, lung, and pancreas in male construction workers: a retrospective cohort study. Lancet.

[B12] Farrow DC, Davis S (1990). Risk of pancreatic cancer in relation to medical history and the use of tobacco, alcohol and coffee. Int J Cancer.

[B13] Muscat JE, Stellman SD, Hoffmann D, Wynder EL (1997). Smoking and pancreatic cancer in men and women. Cancer Epidemiol Biomarkers Prev.

[B14] Alguacil J, Silverman DT (2004). Smokeless and other noncigarette tobacco use and pancreatic cancer: a case-control study based on direct interviews. Cancer Epidemiol Biomarkers Prev.

[B15] Hassan MM, Abbruzzese JL, Bondy ML, Wolff RA, Vauthey J-N, Pisters PW, Evans DB, Khan R, Lenzi R, Jiao L, Li D (2007). Passive smoking and the use of noncigarette tobacco products in association with risk for pancreatic cancer: a case-control study. Cancer.

[B16] Williams RR, Horm JW (1977). Association of cancer sites with tobacco and alcohol consumption and socioeconomic status of patients: interview study from the Third National Cancer Survey. J Natl Cancer Inst.

[B17] Scientific Committee on Emerging and Newly Identified Health Risks (SCENIHR) (2007). Health effects of smokeless tobacco products preliminary report.

[B18] Cogliano V, Straif K, Baan R, Grosse Y, Secretan B, El Ghissassi F (2004). Smokeless tobacco and tobacco-related nitrosamines. Lancet Oncol.

[B19] Winn D, Walrath J, Blot W, Rogot E (1982). Chewing tobacco and snuff in relation to cause of death in a large prospective cohort [Abstract]. Am J Epidemiol.

[B20] Falk RT, Pickle LW, Fontham ET, Correa P, Fraumeni JF (1988). Life-style risk factors for pancreatic cancer in Louisiana: a case-control study. Am J Epidemiol.

[B21] Ghadirian P, Simard A, Baillargeon J (1991). Tobacco, alcohol and coffee and cancer of the pancreas. Cancer.

[B22] US Surgeon General (1986). The health consequences of using smokeless tobacco A report of the Advisory Committee to the Surgeon General, 1986.

[B23] Glover ED, Schroeder KL, Henningfield JE, Severson HH, Christen AG (1989). An interpretative review of smokeless tobacco research in the United States: Part II. J Drug Educ.

[B24] Axéll TE (1993). Oral mucosal changes related to smokeless tobacco usage: research findings in Scandinavia. Eur J Cancer B Oral Oncol.

[B25] Lee PN (2007). Circulatory disease and smokeless tobacco in Western populations: a review of the evidence. Int J Epidemiol.

[B26] Weitkunat R, Sanders E, Lee PN (2007). Meta-analysis of the relation between European and American smokeless tobacco and oral cancer. BMC Public Health.

[B27] Fleiss JL, Gross AJ (1991). Meta-analysis in epidemiology, with special reference to studies of the association between exposure to environmental tobacco smoke and lung cancer: a critique. J Clin Epidemiol.

[B28] Higgins JPT, Thompson SG, Deeks JJ, Altman DG (2003). Measuring inconsistency in meta-analyses. BMJ.

[B29] Bjelke E, Schuman LM (1982). Chewing of tobacco and use of snuff: relationships to cancer of the pancreas and other sites in two prospective studies. Proceedings of the 13th International Congress on Cancer, Seattle, Washington.

[B30] International Agency for Research on Cancer (1985). Tobacco habits other than smoking; betel-quid and areca-nut chewing; and some related nitrosamines [IARC Monographs on the evaluation of the carcinogenic risk of chemicals to humans].

[B31] Heuch I, Kvåle G, Jacobsen BK, Bjelke E (1983). Use of alcohol, tobacco and coffee, and risk of pancreatic cancer. Br J Cancer.

[B32] Schuman LM, Bjelke E, Gibson RW, Watt GD (1982). Cancer among chewers of snuff/tobacco: International cohort comparisons for Norway and the United States (Abstract no. 1172). Proceedings of the XIIIth International Cancer Congress, Seattle, Washington Seattle, Washington.

[B33] Kahn HA, Haenszel W (1966). The Dorn study of smoking and mortality among U.S. veterans: report on eight and one-half years of observation. Epidemiological approaches to the study of cancer and other chronic diseases.

[B34] Rodu B (2007). Snus and the risk of cancer of the mouth, lung, and pancreas [Letter]. Lancet.

[B35] Bolinder G, Alfredsson L, Englund A, de Faire U (1994). Smokeless tobacco use and increased cardiovascular mortality among Swedish construction workers. Am J Public Health.

[B36] International Agency for Research on Cancer (2004). Tobacco smoke and involuntary smoking [IARC Monographs on the evaluation of carcinogenic risks to humans].

[B37] US Surgeon General (2004). The health consequences of smoking A report of the Surgeon General.

[B38] Ramström L (2006). Re: "Smokeless tobacco use and risk of cancer of the pancreas and other organs" by Boffetta *et al *[Letter]. Int J Cancer.

